# A novel hydrophilic fluorescent probe for Cu^2+^ detection and imaging in HeLa cells†

**DOI:** 10.1039/d0ra09894a

**Published:** 2021-03-10

**Authors:** Xinyu Wang, Zhuo Li, Jiaojiao Nie, Liangqiang Wu, Weihong Chen, Shaolong Qi, Hai Xu, Jianshi Du, Yaming Shan, Qingbiao Yang

**Affiliations:** China-Japan Union Hospital of Jilin University Changchun 130033 China dujs@jlu.edu.cn; College of Chemistry, Jilin University Changchun 130021 China yangqb@jlu.edu.cn; Key Laboratory of Lymphatic Surgery Jilin Province, Engineering Laboratory of Lymphatic Surgery Jilin Province Changchun 130033 China; National Engineering Laboratory for AIDS Vaccine, School of Life Sciences, Jilin University Changchun 130012 China shanym@jlu.edu.cn

## Abstract

Copper is an essential element in living systems and plays an important role in human physiology; therefore, methods to detect the concentration of copper ions in living organisms are important. Herein, we report a highly water-soluble naphthalimide-based fluorescent probe that can be used for the detection of Cu^2+^. The probe, BNQ, has high selectivity and sensitivity. The fluorescence intensity of the probe at 520 nm was visible to the naked eye under a UV lamp; upon the gradual addition of Cu^2+^, there was a colour change from green to nearly colourless. Furthermore, the detection limit of BNQ for Cu^2+^ was 45.5 nM. The detection mechanism was investigated using a Job's plot and density functional theory (DFT) calculations. In addition, owing to great biocompatibility, we were able to successfully use BNQ to detect Cu^2+^ in living HeLa cells with low toxicity.

## Abstract 2

Probe BNQ was successfully used for detection of exogenous Cu^2+^ in cells using a rare ESDPT sensing mechanism.

## Graphical abstract

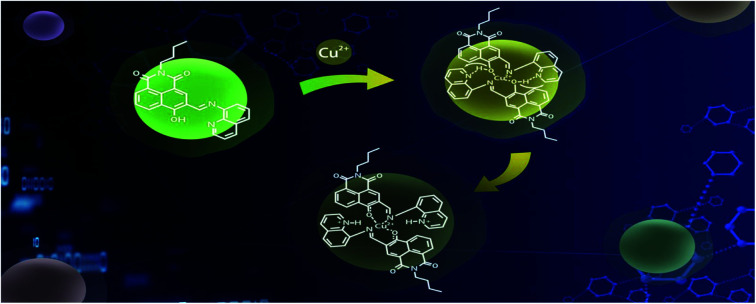

## 1. Introduction

Even when present in trace amounts, copper, a mineral micronutrient, has been associated with a wide range of cellular processes; it plays a vital role as a coenzyme in free-radical scavenging and is involved with mitochondrial respiration, collagen and elastin synthesis, iron metabolism, erythropoiesis, and leukopoiesis.^1–6^ The average concentration of copper in human blood is 100–150 μg dL^−1^ (ref. 46) and the recommended intake of copper for humans is 0.9 mg each day.^7^ Excessive or inadequate intake of copper can cause a variety of diseases.^8,9^ Copper deficiency leads to Menkes disease,^10,11^ haematological neurological sequelae, and complications caused by malabsorption, including macrocytic anaemia, myelopathy, chronic diarrhoea, celiac disease, and inflammatory bowel disease (IBD).^6,12^ Excessive copper causes Wilson disease^13,14^ and copper dyshomeostasis has important implications for Alzheimer's disease pathology.^15–17^ The US Environmental Protection Agency stipulates that the maximum concentration of copper in drinking water should be 1.3 mg L^−1^ (∼1 ppm).^18^ However, because copper has applications in a variety of fields, such as mining, smelting, and fertilising, and due to the abuse of molluscicides and fungicides, humans are exposed to copper *via* contaminants through air, drinking water, soil, and food.^19–23^ It has been reported that copper from drinking water and food can increase its concentration in human blood and accumulate in the kidney, cornea, and brain, which can lead to disease.^24^ For this reason, it is necessary to develop a test method to detect the concentration of copper ions in living organisms.

The main analytical methodologies for detecting Cu^2+^ include atomic absorption spectroscopy, electrochemical sensing, piezoelectric quartz crystal impedance studies, and inductively coupled plasma atomic emission spectrometry.^24–26^ These methods require complicated preparation procedures, expensive instruments and trained professional technicians.^27^ Recently, fluorescent probes have gained attention due to several notable advantages, including high sensitivity, high specificity, good biocompatibility, simplified operation, and minimal invasiveness.^28–31^ Many recent reports describe excellent fluorescent sensors for detecting Cu^2+^.^32–38^ However, there are many unavoidable drawbacks for the probes that have been reported so far, such as weak hydrophilia, tedious synthetic steps, stringent applied conditions, and the likelihood of interference from Hg^2+^, Al^3+^, or Zn^2+^. Therefore, more research is required to develop fluorescent probes with strong hydrophilia, good biocompatibility, low toxicity, high anti-interference ability, and easy preparation.

Herein, we report the design and synthesis of a new fluorescent probe (*E*)-2-butyl-6-hydroxy-5-((quinolin-8-ylimino) methyl)-1*H*-benzo[de]isoquinoline-1,3(2*H*)-dione (BNQ), a naphthalimide Schiff base derivative, to be used for Cu^2+^ detection. The BNQ probe exhibits high selectivity, high sensitivity, low toxicity, and strong hydrophilia, and was successfully used to image Cu^2+^ in living cells. The detection mechanism was based on excited state double-proton transfer (ESDPT), which is not commonly used for Cu^2+^ detection. This has been confirmed with Job's plot analysis and DFT studies. This study may provide a feasible plan for the specific detection of Cu^2+^ in biomedicine and biological pathology.

## 2. Experimental

### 2.1. Materials and instrumentations

All chemicals are commercially available without further purification. 4-Bromo-1-8-naphthalic anhydride, 8-aminoquinoline, ullotropine and other organic reagents were supplied by Shanghai Aladdin Biochemical Technology. The solutions of various testing species were prepared from NaCl, KCl, BaCl_2_, CdCl_2_, MnCl_2_, NiCl_2_, MgCl_2_, HgCl_2_, FeCl_2_, CuCl_2_, ZnCl_2_, FeCl_3_, CrCl_3_, SnCl_4_, Ca(NO_3_)_2_, Pb(NO_3_)_2_, Al(NO_3_)_3_.

^1^H NMR and ^13^C NMR spectra were recorded on a Bruker AVANCE III 400 spectrometer (^1^H, 400 MHz; ^13^C, 101 MHz) in CDCl_3_ or DMSO-d_6_. Fluorescence spectra were obtained on a Hitachi F-4500 fluorescence spectrophotometer (Japan) equipped with a 1 cm quartz cell, and UV-vis spectra were measured using a Hitachi U-3010 spectrometer. Mass spectra were recorded on an Agilent 1290-micr OTOF Q II mass spectrometer (US). The pH of the solution was determined using a Mettler-Toledo instrument. FT-IR spectrum was taken on a Varian Scimitar 1000 spectrometer. All measurements were performed at approximately room temperature.

### 2.2. Synthesis of probe BNQ

**Scheme 1:**
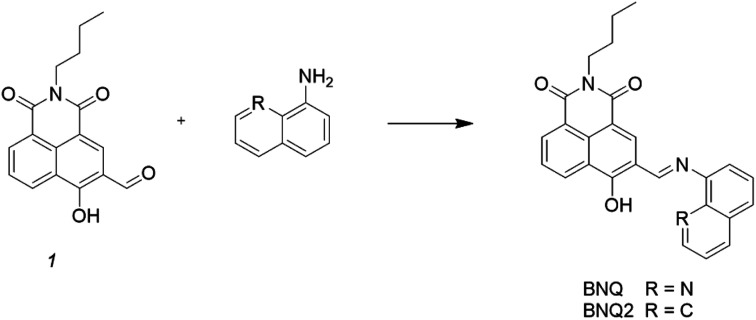
Synthesis of probes BNQ and BNQ2.

The synthesis of the probe is shown in Scheme 1.

Compound 1 (0.1 g, 0.3 mmol) was synthesized according to the literature^39^ and dissolved in methanol (10 mL) along with 8-aminoquinoline (42 mg, 0.3 mmol) in a 50 mL round-bottom flask. The resulting mixture was refluxed for 4 h under a nitrogen atmosphere. After cooling to room temperature, the mixture was filtered through a 0.2 μm nylon membrane filter and rinsed three times with methanol. The solvent was removed under vacuum drying at 50 °C for 12 h, and the crude product was purified by silica gel chromatography to afford pure BNQ as a dark yellow solid. The mp of the product was found to be 260–261 °C. ^1^H NMR (400 MHz, CDCl3) *δ* 15.56 (s, 1H), 9.14 (s, 1H), 8.74 (dd, *J* = 34.8, 9.2 Hz, 2H), 8.55 (d, *J* = 6.5 Hz, 1H), 8.27 (dd, *J* = 42.9, 13.7 Hz, 3H), 7.82–7.71 (m, 2H), 7.60 (d, *J* = 7.9 Hz, 2H), 4.14 (s, 2H), 1.70 (d, *J* = 6.2 Hz, 2H), 1.45 (d, *J* = 6.4 Hz, 2H), 0.98 (t, *J* = 6.7 Hz, 3H). ^13^C NMR (101 MHz, CDCl3) *δ* 182.16 (s), 164.69 (s), 153.30 (s), 151.10 (s), 147.75 (s), 139.79 (s), 136.35 (d, *J* = 11.0 Hz), 133.68 (s), 132.29 (s), 129.17(s), 127.69 (s), 126.90–126.79 (m), 126.64 (d, *J* = 22.8 Hz), 126.14 (s), 123.06 (s), 121.66 (s), 116.35 (s), 113.92 (s), 111.78 (s), 110.78 (s), 110.35 (s), 40.41 (s), 30.65 (s), 20.78 (s), 14.24 (s). HRMS (ESI): *m*/*z* calcd for C_26_H_22_N_3_O_3_ ([M + H]^+^): 424.1656; found 424.1647. FT-IR (KBr) cm^−1^: 3360 (*ν*O–H); 1640 (*ν*C=N). And the structure was characterised by ^1^H NMR, ^13^C NMR, and HRMS as shown in the ESI (Fig. S1–S3†).

The synthesis of probe BNQ2 was similar to that of BNQ, except that 8-aminoquinoline (42 mg, 0.3 mmol) was substituted with naphthalen-1-amine (43 mg, 0.3 mmol). The structure was characterised by ^1^H NMR and ^13^C NMR, as shown in ESI (Fig. S4 and S5†).

### 2.3. Cell culture

The HeLa cells were cultured in DMEM medium containing 10% fetal bovine serum at 37 °C in a 95% humidity under a 5% CO_2_ environment. Approximately 24 h later, the cells adhered to the surface of the dish. The cells that exhibited the logarithmic growth phase were selected for the following experiments.

### 2.4. Cytotoxicity of BNQ probe

To evaluate the cytotoxicity of the BNQ probe, the cell viability of the HeLa cells was investigated using the standard MTT (3-(4,5-dimethyl-2-thiazolyl)-2,5-diphenyl-tetrazolium bromide) assay. The HeLa cells were incubated with 0, 0.0625, 0.125, 0.25, 0.5, and 1 μM of the BNQ probe for 24 h at 37 °C. Subsequently, 10 μL MTT (5 mg mL^−1^) prepared in HEPES was added and coincubated for another 4 h. We used the enzyme labeller to read the OD value at 490 nm and calculated the cell survival rate according to the following equation:Cell viability (%) = OD_490_ (sample)/OD_490_ (control) × 100%

### 2.5. Cell imaging of BNQ probe

For cell imaging, Hela cells were seeded in 24-well plates (2 × 10^5^ cells per well) and allowed to adhere for 24 h. The cells were incubated with 1 μM probe for 30 min, and then treated with 10 μM Cu^2+^ for another 30 min. Confocal microscopic imaging was performed before and after 30 min treated with Cu^2+^ under excitation at 520 nm.

## 3. Results and discussion

### 3.1. Effect of pH

**Fig. 1:**
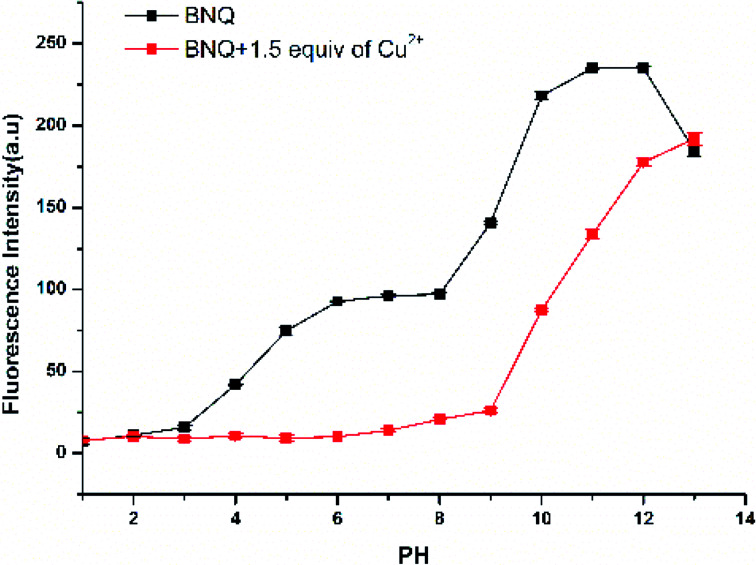
Fluorescence intensity of BNQ (5 μM) with and without Cu^2+^ in solutions of different pH. Test condition: Cu^2+^ (10 μM), DMSO/HEPES (1/9, v/v), *λ*_ex_ = 390 nm, slits: 2.5/2.5.

The effect of pH on the emission intensity of BNQ and BNQ-Cu^2+^ was measured (Fig. 1). When the pH was lower than 4, the fluorescence intensity of the BNQ probe was minimal, which may be due to the reaction of hydrogen ions in the solution with the nitrogen on the probe. The fluorescence intensity increased gradually from pH 6.0 to 8.0 and then rapidly increased from pH 8.0 to 10.0. After the addition of Cu^2+^, the fluorescence intensity decreased gradually from pH 4.0 to 12.0, possibly due to the reaction of the hydroxide ions in the solution with the phenolic hydroxyl group. However, the fluorescence intensity remained high when the pH was greater than 9.0 because of the reaction of the hydroxide with Cu^2+^ to Cu(OH)_2_. Therefore, the pH range 6.0–8.0 was considered optimal, because the fluorescence of the BNQ probe was stable, and there was a significant change in fluorescence upon adding Cu^2+^ in this range. pH = 6.0 was selected as the test condition of this experiment because the fluorescence reduction was most significant.

### 3.2. Probe response time to Cu^2+^

**Fig. 2:**
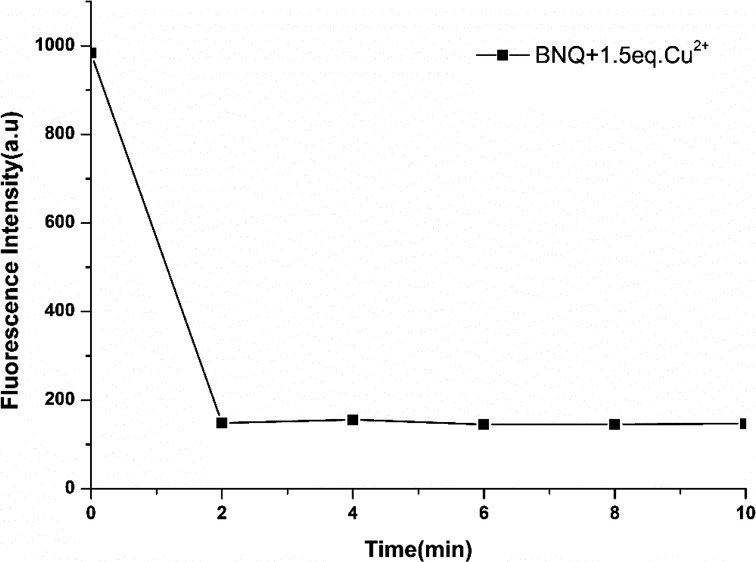
Probe response time to Cu^2+^. *λ*_ex_ = 390 nm, *λ*_em_ = 520 nm.

Subsequently, time-dependent fluorescence intensity studies were carried out for Cu^2+^ (10 μM) determination by BNQ (5 μM) in DMSO/Tris (1/9, v/v) (Fig. 2). After adding Cu^2+^, the fluorescence intensity of BNQ dropped sharply within the first 2 min and then reached saturation, which indicated that the probe reacted with Cu^2+^ completely in 2 min.

### 3.3. Fluorescent response of BNQ probe in the presence of Cu^2+^

We carried out UV-vis absorption studies on the BNQ probe in the presence of Cu^2+^ (5 μM, DMSO/HEPES = 1/9, v/v, pH = 6.0). With increasing Cu^2+^ in the solution, the absorption peak between 300 nm and 500 nm gradually increased (Fig. S6†). The UV-vis absorption remained constant after 1.5 equivalents Cu^2+^ were added to the solution. Along with the spectral changes, a notable colour change from bright yellow to colourless was easily observed by the naked eye (Fig. S8†).

**Fig. 3:**
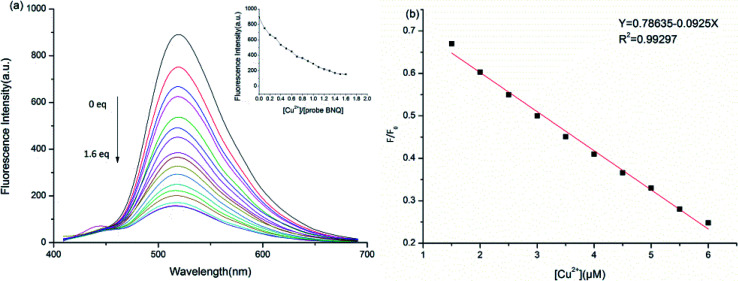
(a) Fluorescence titration spectra of BNQ (5 μM) in DMSO/HEPES buffer (10 mM, pH = 6.0, 1/9, v/v) with the addition of Cu^2+^; (b) The fluorescence intensity ratio (*F*/*F*_0_) of BNQ is almost linearly related to the concentration of Cu^2+^ in range of 0–7.5 μM.

To further understand the sensing properties of the BNQ probe for Cu^2+^ detection, fluorescence titration was performed by increasing the concentration of Cu^2+^ in a DMSO/HEPES (1/9, v/v) solution. As shown in Fig. 3a, with the increase in Cu^2+^, the fluorescence decreased gradually from 520 nm and reached the minimum when 1.5 equivalents of Cu^2+^ were added. The colour changed from green to colourless under a UV lamp, which indicated that Cu^2+^ can be detected because of the complexation of phenolic hydroxyl and imide structures on the BNQ probe with copper ions. The regression equation *y* = 0.78635–0.0925*x* was obtained from the fluorescence titration data, and a good linear relationship (*R*^2^ = 0.99297) between the fluorescence intensity and Cu^2+^ concentration was observed (Fig. 3b). The detection limit of the BNQ probe was calculated to be 45.5 nM based on the following equation:^40^DL = *KS*_bl_/*S*where *K* = 3; *S*_bl_ is the standard deviation of the blank solution; and *S* is the slope of the calibration curve. The limit of detection (LOD) for Cu^2+^ was calculated to be 45.5 nM, which is significantly lower than that for Cu^2+^ limit (20.5 μM) set by the World Health Organization.^41^

### 3.4. Fluorescence response to various metal ions

**Fig. 4:**
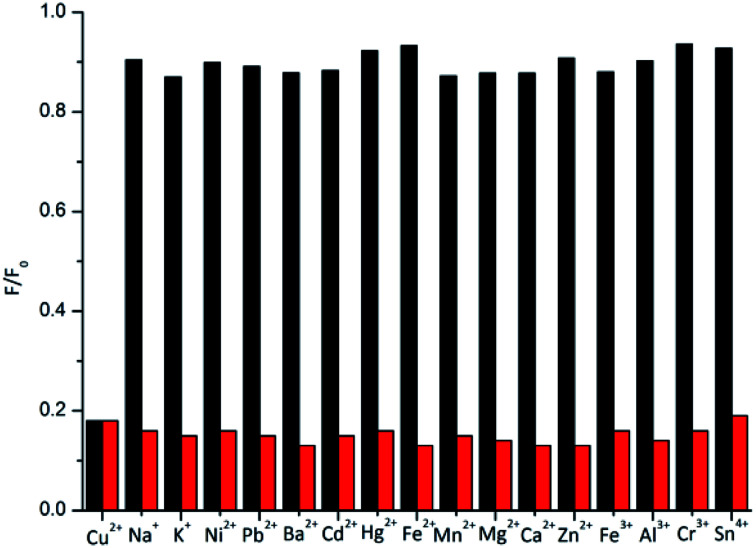
The variation in the fluorescence ratio (F/F0) of BNQ (5 μM) after adding 15 equiv. of different metal cations (black bar) and after adding 1.5 equiv. Cu2+ in the presence of 15 equiv. of various cations (red bars). (*λ*_ex_ = 390 nm, *λ*_em_ = 520 nm, slits: 10/5, DMSO/HEPES = 1/9).

As shown in Fig. 4, we also performed selectivity experiments and anti-interference ability experiments for the BNQ probe. A high fluorescence intensity of the BNQ probe (5 μM) at 520 nm was detected immediately before the addition of any metal ions, and the fluorescence spectrum did not change significantly even after the addition of 15 equivalents of other metal ions (Ni^2+^, Mn^2+^, Mg^2+^, Fe^2+^, Pb^2+^, Ca^2+^, Ba^2+^, Zn^2+^, Cd^2+^, Hg^2+^, Sn^4+^, Cr^3+^, Fe^3+^, Al^3+^, Na^+^ and K^+^) (Fig. S9 and S10a†), However, after adding Cu^2+^ (1.5 equivalents) to the mixed solutions of the BNQ probe (5 μM) and 15 equivalents of every interference metal ion mentioned above, the fluorescence intensity decreased significantly, changing from green to colourless when observed under the UV lamp (Fig. S10b†). The results demonstrated that the BNQ probe can detect Cu^2+^ with excellent selectivity and anti-interference ability.

### 3.5. Possible mechanism

**Scheme 2:**
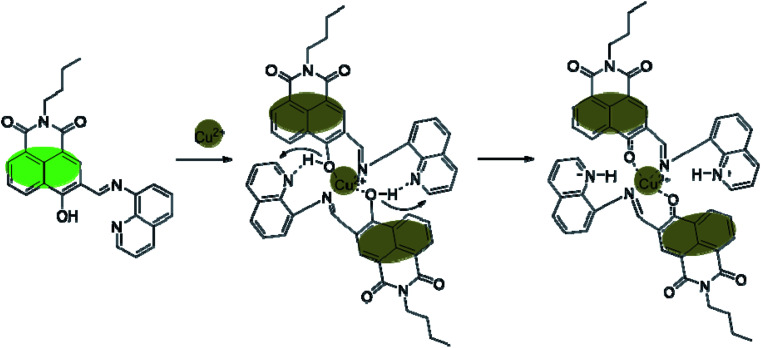
Proposed binding mechanism between the receptor BNQ and Cu^2+^.

The stoichiometry of the obtained BNQ–Cu^2+^ complex was determined to be 2 : 1 (BNQ : Cu^2+^) using Job's method and the Benesi–Hildebrand method (Fig. S11 and S12†). And according to previous literature reports,^42,43^ Cu^2+^ is a powerful fluorescence emission quencher due to the paramagnetism. In addition, C=O and C=N groups can be involved in complexation with Cu^2+^. Thus, we hypothesised that the reaction mechanism would proceed as follows (Scheme 2): an unstable seven-membered ring forms through hydrogen bonding after the phenolic hydroxyl oxygen on the naphthalimide interacts with the imine nitrogen and Cu^2+^. Subsequently, the steric hindrance of the molecule decreases and excited state intramolecular proton transfer (ESIPT) occurs. The hydrogen transfers from the phenolic hydroxyl group to the quinoline nitrogen. These changes disrupt the conjugated structure of naphthalimide, leading to fluorescence quenching. In addition, the geometric configuration of the BNQ probe implies that it more easily coordinates with Cu^2+^ rather than the other ions due to the different ionic radius. To further study the effect of nitrogen in quinoline on the reaction mechanism, BNQ2 was synthesised and its anti-interference ability was studied. The results (Fig. S13†) showed that the absence of nitrogen on the naphthalene ring resulted in lower selectivity of the probe.

### 3.6. Theoretical calculation

**Fig. 5:**
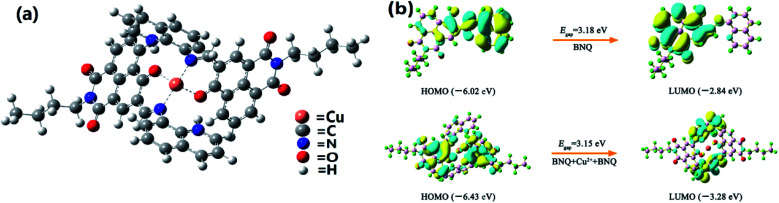
(a) DFT optimised structures of BNQ–Cu^2+^–BNQ complex. (b) Molecular orbital profiles of BNQ and BNQ–Cu^2+^–BNQ molecules.

Density functional theory (DFT) calculations were performed using the Gaussian 16 program^44,45^ to understand the complexation structures and electronic properties of BNQ + Cu^2+^ (Fig. 5). The DFT optimisation of each structure was conducted with the CAM-B3LYP functional and 6-31G(d)/Lanl2dz basis sets. For Cu, we used the LANL2DZ basis set, and treated all other atoms using the 6-31G(d) basis set. As shown in Fig. 5a, we suggest that there are four binding sites for each dimer, which involve imine N and hydroxyl O atoms coordinated to one Cu^2+^ ion. The hydrogens from the hydroxyl phenols were transferred to nitrous oxide by intramolecular H-bonding simultaneously. The highest occupied molecular orbital (HOMO) and lowest unoccupied molecular orbital (LUMO) of the BNQ probe and BNQ–Cu^2+^–BNQ molecule are as shown in Fig. 4b. The HOMO–LUMO gap (*E*_gap_) of the BNQ–Cu^2+^–BNQ molecule was 3.15 eV, slightly lower than that of the BNQ probe (3.18 eV), although it is still stable.

### 3.7. Biological research

Before cell imaging, the cytotoxicity of BNQ was investigated to ensure safe application in living organisms. As shown in Fig. S15,† the cell viability was maintained above 90% after incubation with different concentrations of BNQ/Cu^2+^/BNQ + Cu^2+^at 37 °C for 4 h (DMSO/HEPES = 1/9, v/v, pH = 6.0), indicating that the BNQ probe has good biocompatibility and low cytotoxicity in cells.

**Fig. 6:**
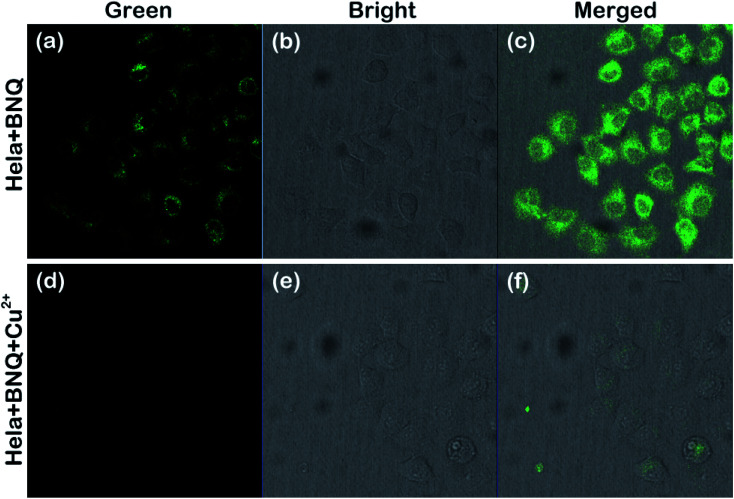
Fluorescence images (a and d), bright-field transmission images (b and e) and merged images (c and f) of HeLa cells incubated with the BNQ probe (1 μM) initially and 30 min after treatment with Cu^2+^ (10 μM, DMSO/HEPES = 1/9, v/v, pH = 6.0). *λ*_em_ = 515 nm.

Subsequently, we further examined the ability of the probe to monitor Cu^2+^ in HeLa cells. As shown in Fig. 6, a strong green fluorescence could be observed when HeLa cells were only treated with 1 μM of the BNQ probe. The fluorescence disappeared after subsequent treatment with 10 μM of Cu^2+^ for another 30 min, indicating that the BNQ probe can monitor Cu^2+^ in living cells with excellent membrane permeability.

### 3.8. Comparison with previous probes

The method for detecting Cu^2+^ proposed in this work was compared with other previous works.^32–38^ As shown in Table 1, the probe BNQ not only has a relatively lower detection limit, but also can detect Cu^2+^ with shorter time and better water solubility. Therefore, the probe BNQ is a promising candidate to detect Cu^2+^*in vivo* and *in vitro*. In addition, the probe BNQ detects Cu^2+^ using a rather rare mechanism of ESDPT.

**Table 1:** Comparison of the present work with other previous works for Cu^2+^ detection

Probe structure	Solvent system (v/v)	Detection limit (M)	Time	pH	Application	Mechanism	Ref.
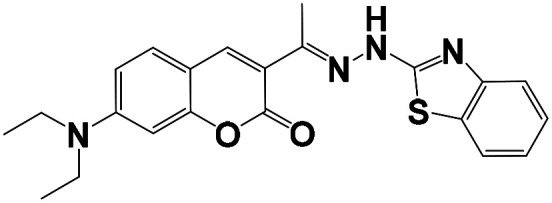	CH_3_CN/HEPES = 1/1	5.8 × 10^−8^	1 h	3–8	MCF-7 cells	The chelation-controlled C=N isomerization in anhydrous acetonitrile and Cu^2+^-promoted cyclization reaction in aqueous acetonitrile	32
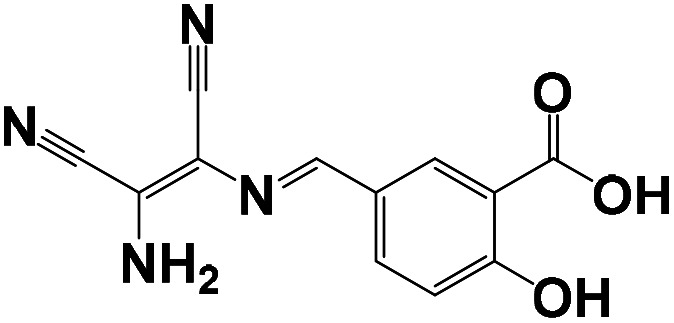	CH_3_CN/HEPES = 1/9	2.19 × 10^−7^	11 min	7.0	HepG2 cells	Molecular conjugation enlarged after coordination with Cu^2+^ ions	33
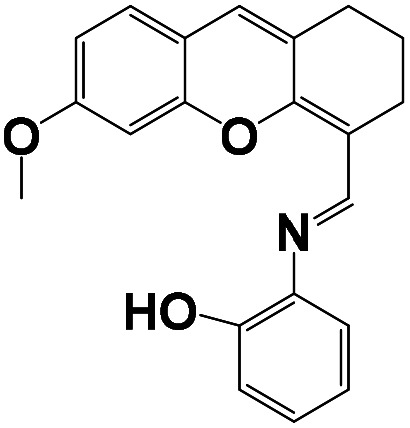	CH_3_CN/HEPES = 4/1	3.30 × 10^−5^	—	4–9	HepG2 cells	ICT	34
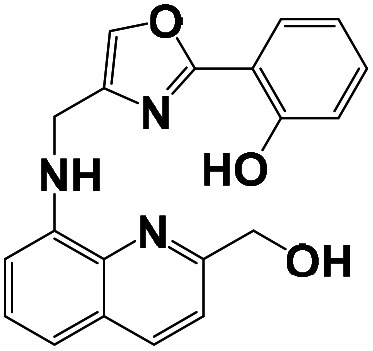	DMSO/Tris = 1/1	2.14 × 10^−8^	—	4–11	PC-12 cells	ICT	35
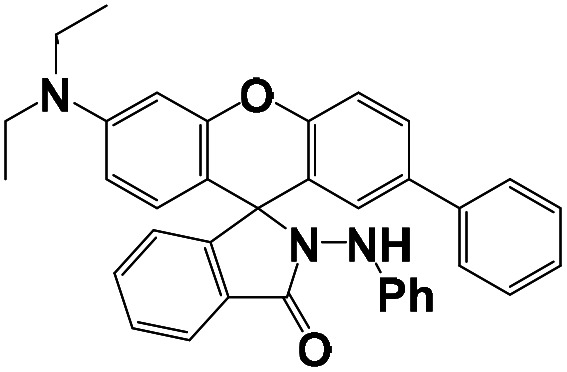	CH_3_CN/HEPES = 1/1	3.54 × 10^−8^	30 min	5–9	Hela cells	Carbon–oxygen bonds break, identification groups fall off, probe fluorescence recovery	36
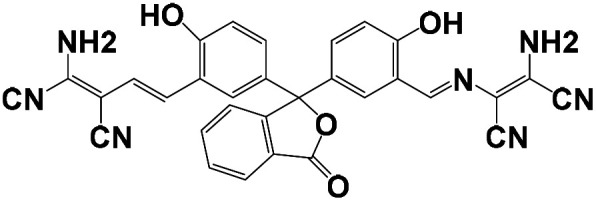	EtOH/H_2_O = 9/1	2.81 × 10^−6^	—	—	Water samples	ESIPT	37
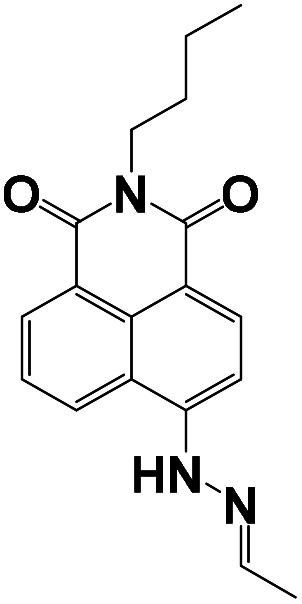	CH_3_CN/HEPES = 4/1	3.20 × 10^−7^	20 min	2–12	293 T cells	Carbon–oxygen bonds break, identification groups fall off, probe fluorescence recovery	38
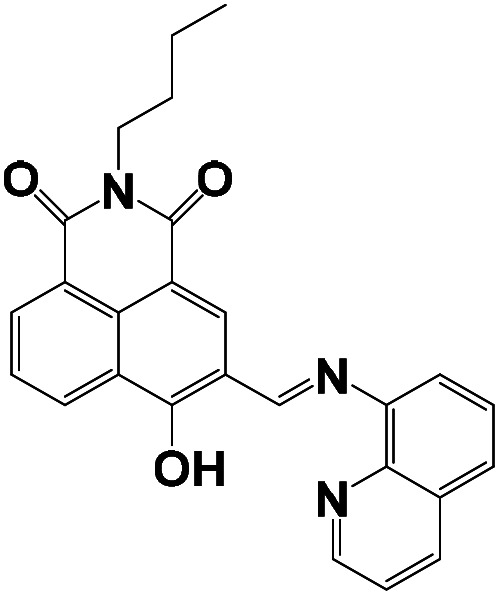	DMSO/HEPES = 1/9	4.55 × 10^−8^	2 min	6–8	Hela cells	ESDPT	This work

## 4. Conclusions

In summary, we have designed and synthesised a naphthalimide-derived fluorescence probe with high selectivity, excellent hydrophilia, and a quick response time, to be used for the detection of Cu^2+^. The detection limit of the BNQ probe was 45.5 nM. The mechanism is based on the ESDPT reaction of naphthalimide in which hydrogen transfers from the phenolic hydroxyl group to the quinoline nitrogen twice after reaction with Cu^2+^. As a result, the fluorescence of the BNQ probe was quenched. During detection, our hypothesised BNQ–Cu^2+^–BNQ model was confirmed by Job's plot analysis and DFT studies. In addition, the BNQ probe was successfully used to image Cu^2+^ in living cells, demonstrating its potential as a tool for monitoring trace Cu^2+^ in biological and pathological processes.

## Conflicts of interest

There are no conflicts to declare.

## Supplementary Material

RA-011-D0RA09894A-s001

## References

[cit1] Domaille D. W., Que E. L., Chang C. J. (2008). Nat. Chem. Biol..

[cit2] Zhou J., Liu C., Francis M., Sun Y., Ryu M.-S., Grider A., Ye K. (2020). Nutrients.

[cit3] Waldron K. J., Rutherford J. C., Ford D., Robinson N. J. (2009). Nature.

[cit4] Gletsu-Miller N., Wright B. N. (2013). Adv. Nutr..

[cit5] Kumar N., Ahlskog J. E., Gross Jr J. B. (2004). Clin. Gastroenterol. Hepatol..

[cit6] Balaet C., Coculescu B. I., Balaet M., Manole G., Dinca G. V. (2018). J. Enzyme Inhib. Med. Chem..

[cit7] Institute of Medicine , Dietary Reference Intakes: The Essential Guide to Nutrient Requirements, The National Academies Press, Washington, DC, 2006

[cit8] Jomova K., Valko M. (2011). Toxicology.

[cit9] Vetlenyi E., Racz G. (2020). Orv. Hetil..

[cit10] Kaler S. G. (2011). Nat. Rev. Neurol..

[cit11] Ren J., Wang S., Li Y., Yang Q., Song Y., Li Y. (2018). Gaodeng Xuexiao Huaxue Xuebao/Chemical Journal of Chinese Universities.

[cit12] Griffith D. P., Liff D. A., Ziegler T. R., Esper G. J., Winton E. F. (2009). Obesity.

[cit13] Cox D. W., Moore S. D. P. (2002). J. Bioenerg. Biomembr..

[cit14] Wentworth B. J., Stotts M. (2020). Practical Gastroenterology.

[cit15] Lovell M. A., Robertson J. D., Teesdale W. J., Campbell J. L., Markesbery W. R. (1998). J. Neurol. Sci..

[cit16] Ejaz H. W., Wang W., Lang M. (2020). Int. J. Mol. Sci..

[cit17] Gromadzka G., Tarnacka B., Flaga A., Adamczyk A. (2020). Int. J. Mol. Sci..

[cit18] U. EPA , National Primary Drinking Water Regulations, 816-F-809-004, 2009

[cit19] Wei B. G., Yang L. S. (2010). Microchem. J..

[cit20] Li Z. Y., Ma Z. W., van der Kuijp T. J., Yuan Z. W., Huang L. (2014). Sci. Total Environ..

[cit21] Manta D. S., Angelone M., Bellanca A., Neri R., Sprovieri M. (2002). Sci. Total Environ..

[cit22] Robinson B. H. (2009). Sci. Total Environ..

[cit23] Du B. Y., Zhou J., Lu B. X., Zhang C., Li D. M., Zhou J., Jiao S. J., Zhao K. Q., Zhang H. H. (2020). Sci. Total Environ..

[cit24] Quang D. T., Kim J. S. (2010). Chem. Rev..

[cit25] Zhou X., Wu X., He H., Liang H., Yang X., Nie J., Zhang W., Du B., Wang X. (2020). Sens. Actuators, B.

[cit26] Hao C., Guo X., Lai Q., Li Y., Fan B., Zeng G., He Z., Wu J. (2020). Inorg. Chim. Acta.

[cit27] Ye F., Chai Q., Xiao-Min L., Ming-Qiang L., Wang Z.-Q., Fu Y. (2017). Molecules.

[cit28] Chan J., Dodani S. C., Chang C. J. (2012). Nat. Chem..

[cit29] Wang J. J., Guo J., Dou L. L., Wang R., Song Y., Yang Q. B., Du J. S., Li Y. X. (2019). Chem. Res. Chin. Univ..

[cit30] Wang J., Qi S., Du J., Yang Q., Song Y., Li Y. (2019). Chem. Res. Chin. Univ..

[cit31] Li Z., Xu Y., Xu H., Cui M., Liu T., Ren X., Sun J., Deng D., Gu Y., Wang P. (2021). Spectrochim. Acta, Part A.

[cit32] Zhang Z., Liu Y., Wang E. (2019). Dyes Pigm..

[cit33] Jiang N., Gong X., Zhong T., Zheng Y., Wang G. (2020). J. Mol. Struct..

[cit34] Li B., Kou J., Mei H., Gu X., Wang M., Xie X., Xu K. (2020). Anal. Methods.

[cit35] Wang P., Yao K., Fu J., Chang Y., Li B., Xu K. (2019). Spectrochim. Acta, Part A.

[cit36] Qiu Q., Yu B., Huang K., Qin D. B. (2020). J. Fluoresc..

[cit37] Erdemir S., Malkondu S. (2019). Dyes Pigm..

[cit38] Fu Y., Pang X.-X., Wang Z.-Q., Chai Q., Ye F. (2019). Spectrochim. Acta, Part A.

[cit39] zhang H., Yin C., Liu T., Chao J., Zhang Y., Huo F. (2017). Dyes Pigm..

[cit40] Liu D., Wang Y., Wang R., Wang B., Chang H., Chen J., Yang G., He H. (2018). Inorg. Chem. Commun..

[cit41] Kang J. H., Lee S. Y., Ahn H. M., Kim C. (2016). Inorg. Chem. Commun..

[cit42] Shen K., Mao S., Shi X., Aderinto S. O., Xu Y., Wu H. (2018). J. Appl. Spectrosc..

[cit43] Xie P., Zhu Y., Huang X., Gao G., Wei F., Guo F., Jiang S., Wang C. (2019). Spectrochim. Acta, Part A.

[cit44] Bauernschmitt R., Ahlrichs R. (1996). Chem. Phys. Lett..

[cit45] Scalmani G., Frisch M. J., Mennucci B., Tomasi J., Cammi R., Barone V. (2006). J. Chem. Phys..

[cit46] Ali Hassan Refat H., Hassan A. I., Hassan Y. F., El-Wekil M. M. (2020). Anal. Bioanal. Chem..

