# Supplementary material for: A novel hydrophilic fluorescent probe for Cu2+ detection and imaging in HeLa cells
Source: RSC Adv. 2021 Mar 10;11(17):10264–71. doi: 10.1039/d0ra09894a (PMC8695708; doi:10.1039/d0ra09894a)
Supplement: RA-011-D0RA09894A-s001 [file RA-011-D0RA09894A-s001.pdf]

Electronic Supplementary Material (ESI) for RSC Advances.
This journal is © The Royal Society of Chemistry 2021

Electronic Supplementary Material (ESI) for RSC Advances

This journal is © The Royal Society of Chemistry 2020

Supporting Information

A novel hydrophilic fluorescent probe for Cu^{2+} detection and imaging in Hela cells

Xinyu Wang, [‡,a, b, c] Zhuo Li, [‡, b,c] Jiaojiao Nie,[d] Liangqiang Wu,[b,c] Weihong Chen,[b] Shaolong Qi,[a,c] Hai Xu,[b] Jianshi Du,[*,a,c] Yaming Shan[*,d] and Qingbiao Yang,[*,b,c]

a. China-Japan Union Hospital of Jilin University, Changchun 130033, China.

b. College of Chemistry, Jilin University, Changchun, 130021, China.

c. Key Laboratory of Lymphatic Surgery Jilin Province, Engineering Laboratory of Lymphatic Surgery Jilin Province, Changchun, 130033, China.

d. National Engineering Laboratory for AIDS Vaccine, School of Life Sciences, Jilin University, Changchun 130021, China.

‡ These two authors equally contribute to this paper

Correspondence to: Jianshi Du (E-mail:dujs@jlu.edu.cn) Yaming Shan (E-mail: shanym@jlu.edu.cn) and Qingbiao Yang (E-mail: yangqb@jlu.edu.cn)

Table of contents

Page

The probe BNQ performance investigation…. …………. … ……… ..……..…...…...….S3

Stability Constant….…… … …………. …………..….…....….…....……...…….…......….S3

The probe BNQ2 performance investigation….……..…. ………..… ……...…...…….S3

Synthesis of Sensor BNQ2….…………. …………..….…....….…....……...…….…......….S3

Selectivity and Competition Studies.…….….…….….…….….……….…..….…..……...….…..…….S3

Figures ………..………..………..………..………..….…....….…....……...…….…......….S4

The probe BNQ performance investigation

Stability Constant

The stability constant for probe BNQ-Cu^{2+}-BNQ was calculated from the Benesi-Hildebrand plots based on the standard equation: $1/(F-F_0) = (1/\{Ks(F_{max}-F_0)[Cu^{2+}]^{1/2}\}+1/(F_{max}-F_0)$, where F_0 is the absorption of probe BNQ in the absence of Cu^{2+}, and $(F-F_0)$ is the discrepancy of absorption between the absence and presence of Cu^{2+}; $[Cu^{2+}]$ is the concentration of Cu^{2+} (Figure S12). The stability constant Ks was given by the ratio of intercept/slop, Ks = 3.418×10^5 (R^2=0.98438) using the linear analysis.

The probe BNQ2 performance investigation

Synthesis of Sensor BNQ2

The synthetic route for probe BNQ2 was displayed in Scheme 1. The product BNQ2 was synthesized in 78% yield by the condensation reaction of compound *1* with naphthylamine in MeOH under reflux for 4 h. ^{1}H NMR (400 MHz, DMSO) δ 15.89 (s, 1H), 9.56 (s, 1H), 8.66 (s, 1H), 8.54 (s, 1H), 8.44 (s, 1H), 8.19 (s, 1H), 8.09 (s, 1H), 7.99 (s, 2H), 7.81 (s, 1H), 7.70 (s, 3H), 4.00 (s, 2H), 1.59 (s, 2H), 1.35 (d, J = 7.0 Hz, 2H), 0.93 (s, 3H). ^{13}C NMR (101 MHz, $CDCl_3$) δ 164.32 (s), 163.61 (s), 162.04 (s), 160.64 (d, *J* = 3.0 Hz), 158.00 (s), 137.85 (s), 136.05 (dd, *J* = 5.8, 4.3 Hz), 134.29 – 134.18 (m), 133.63 (s), 132.30 (s), 131.33 (s), 128.67 (s), 128.41 (s), 127.90 (s), 127.72 (s), 127.34 (s), 126.05 (s), 125.72 (s), 122.76 (s), 121.14 (s), 114.81 (s), 111.61 (s), 111.09 (s), 40.16 (s), 30.30 (s), 20.45 (s), 13.90 (s). (Figure S4-S5).

Selectivity and Competition Studies

in order to observe the selectivity of BNQ2 to Cu^{2+}. We plotted the histogram of BNQ2. As shown as Fig. S13. Black means interference, red means competition.

Figures

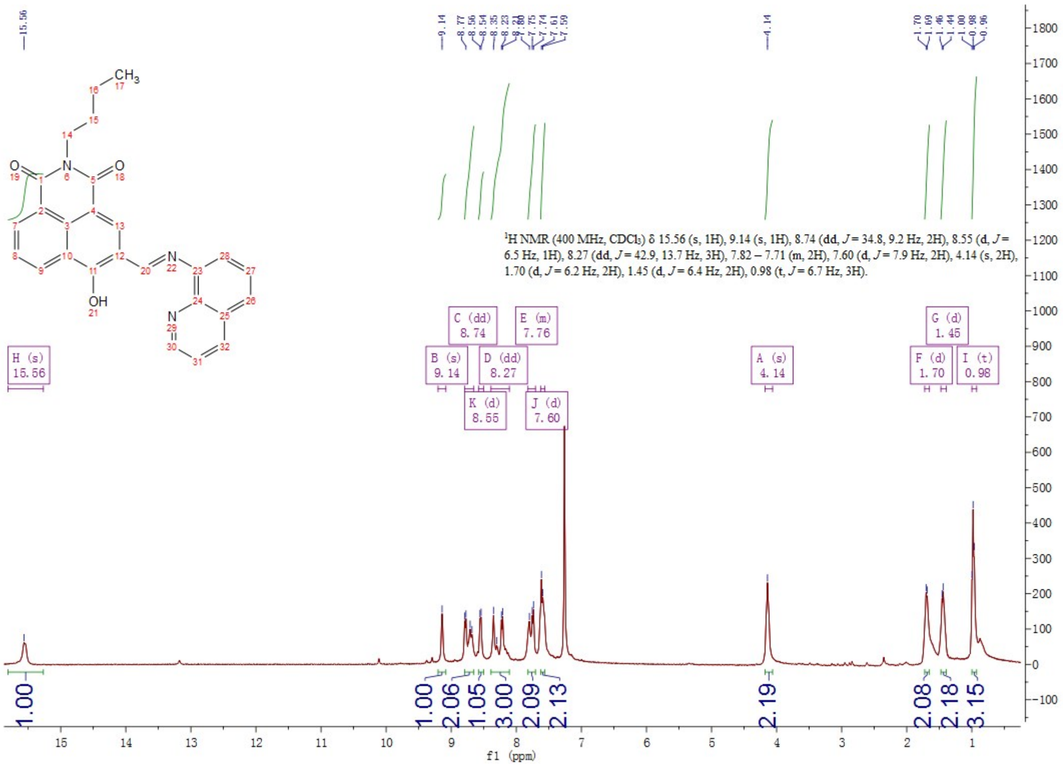

Fig. S1 ^1H NMR spectra of BNQ.

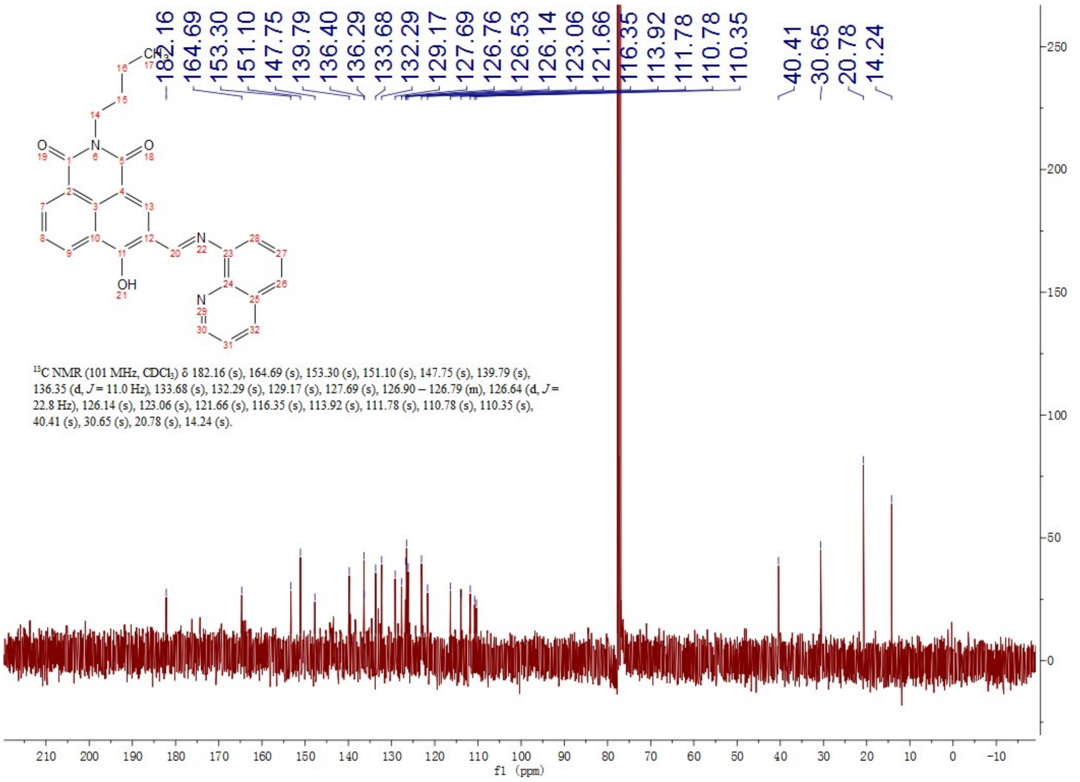

Fig. S2 ^{13}C NMR spectra of BNQ.

Acquisition Parameter

Source Type	ESI	Ion Polarity	Positive	Set Nebulizer	0.4 Bar
Focus	Active	Set Capillary	4500 V	Set Dry Heater	200 °C
Scan Begin	50 m/z	Set End Plate Offset	-500 V	Set Dry Gas	5.0 l/min
Scan End	1200 m/z	Set Collision Cell RF	100.0 Vpp	Set Divert Valve	Waste

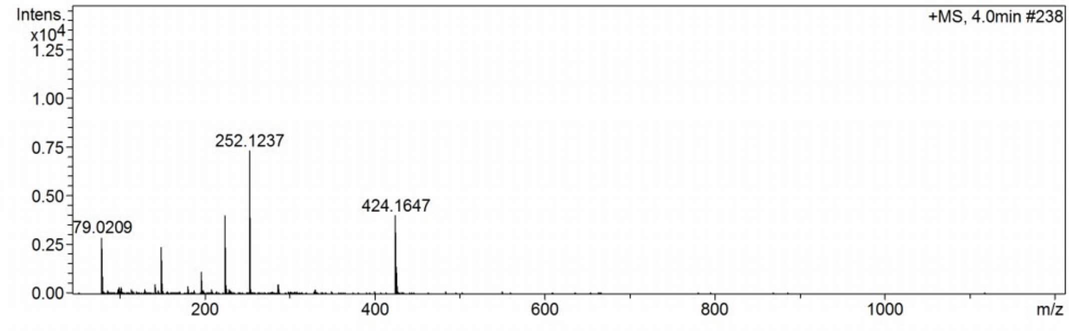

Meas. m/z	#	Formula	m/z	err [ppm]	Mean err [ppm]	rdb	N-Rule	e¡¥ Conf	mSigma	Std I	Std Mean m/z	Std I VarNorm	Std m/z Diff	Std Comb Dev
424.1647														
	1	C 26 H 22 N 3 O 3	424.1656	2.1	4.3	17.5	ok	even	32.47	0.0436	0.0027	0.0192	0.0025	0.8427

Fig. S3 HRMS spectra of BNQ.

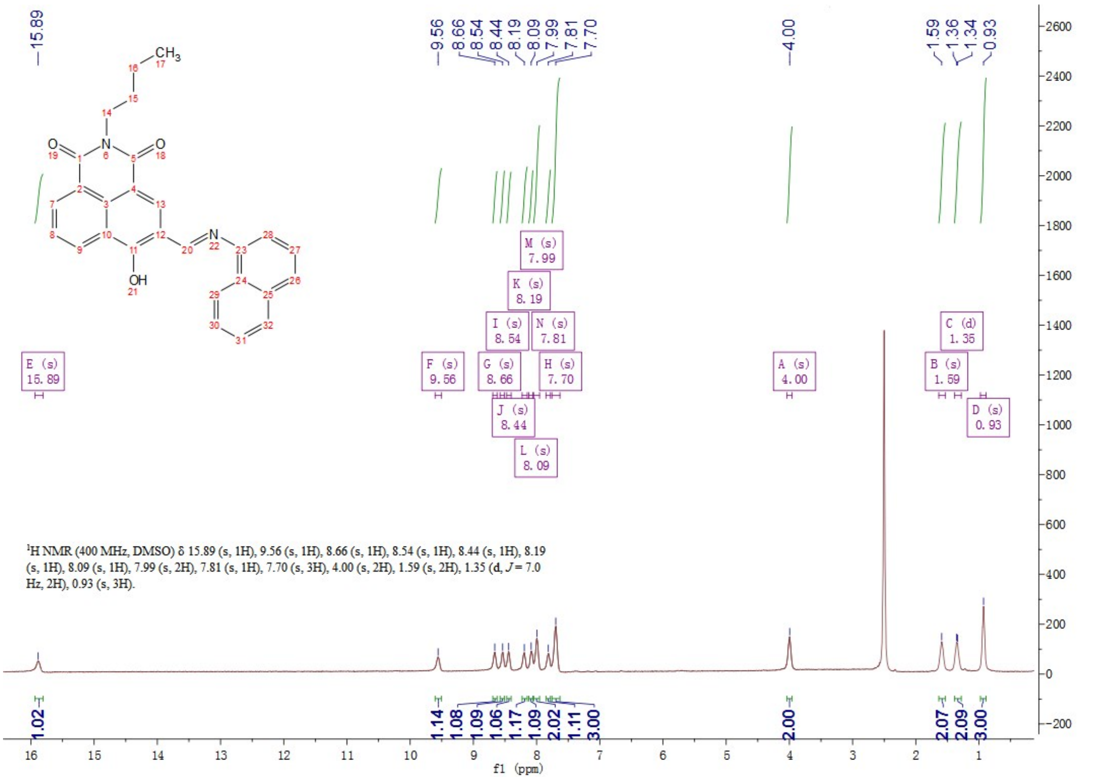

Fig. S4 ^{1}H NMR spectra of BNQ2.

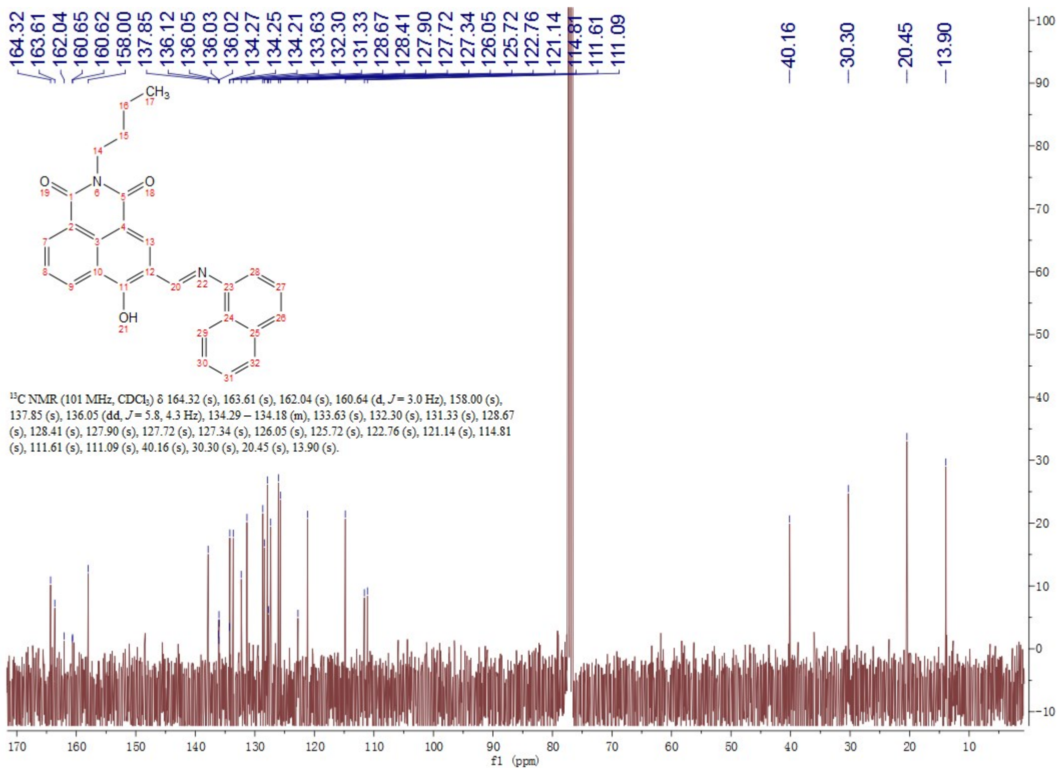

Fig. S5 ^{13}C NMR spectra of BNQ2.

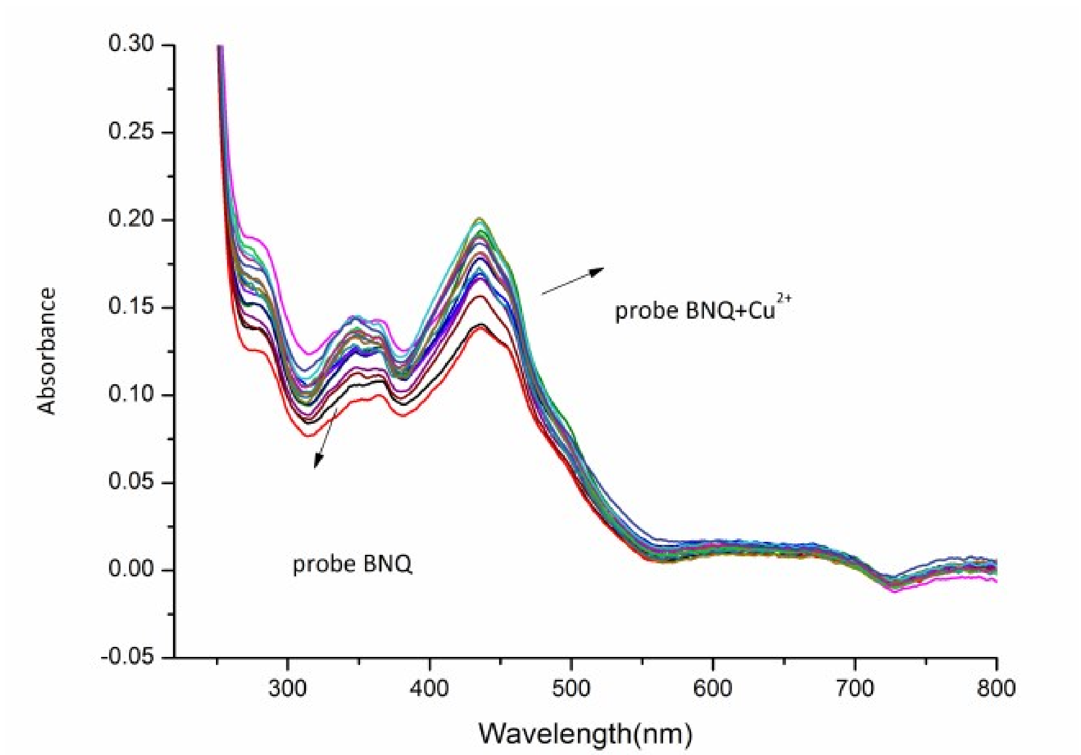

Figure S6. UV-vis absorption spectroscopy of BNQ (5 μM) in DMSO/HEPES buffer (10 mM, pH = 6.0, 1/9, v/v) with addition of Cu^{2+}

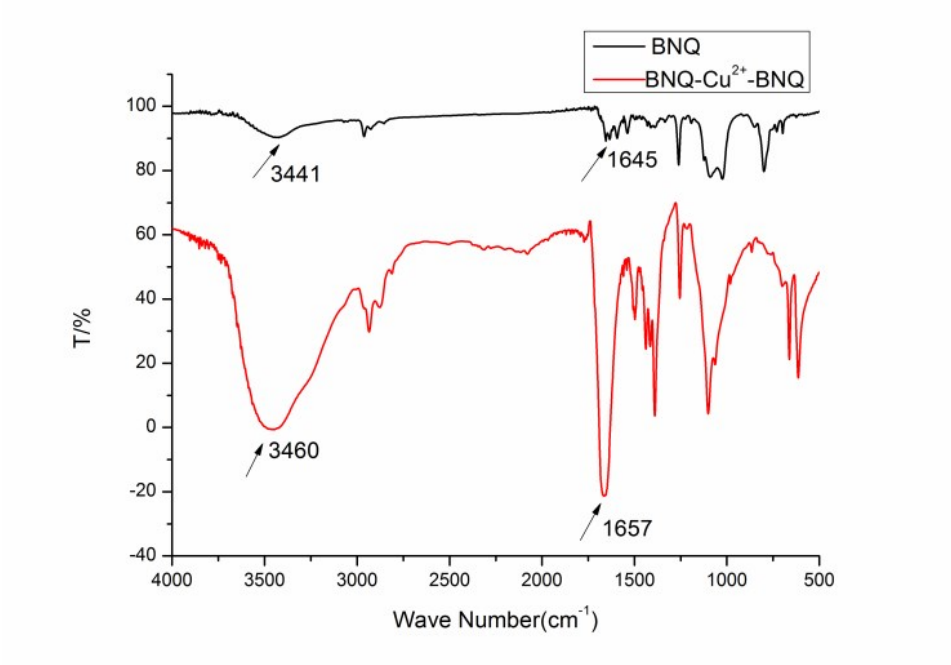

Fig. S7 IR spectra of BNQ and BNQ-Cu^{2+}-BNQ

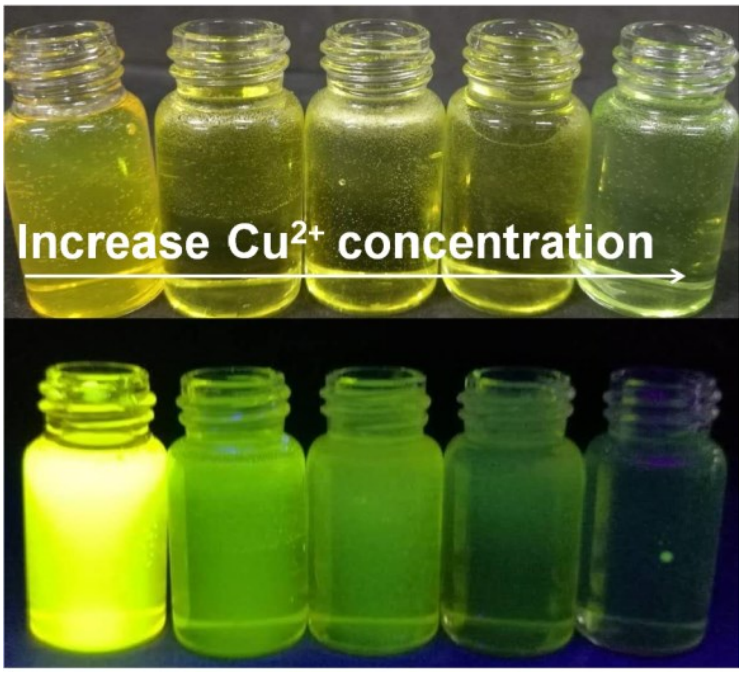

Fig. S8 As the concentration of Cu^{2+} increases, the color of the probe BNQ changes under sunlight and the UV lamp.

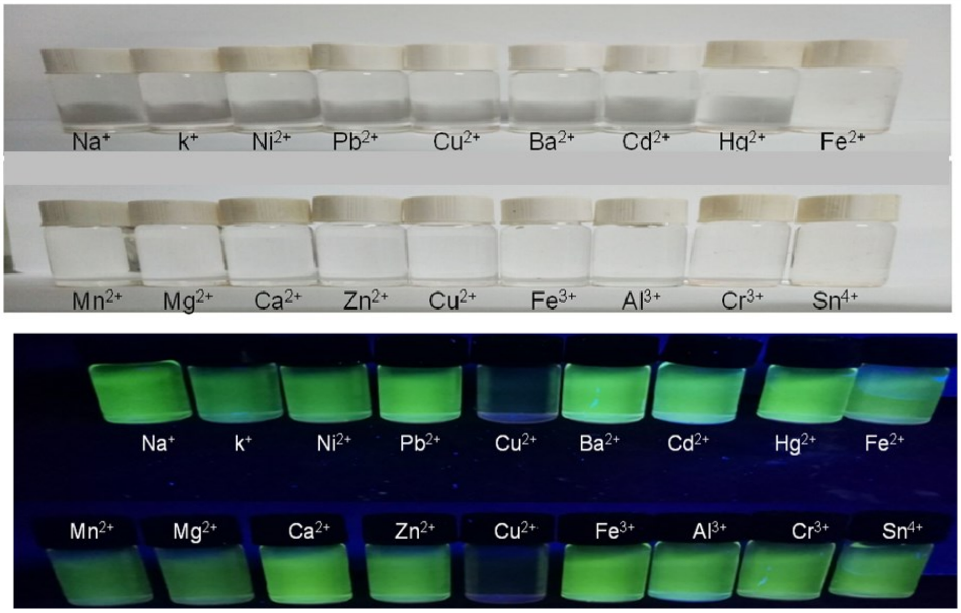

Fig. S9 Photographs of color changes and fluorescence responses of BNQ (5μM) upon addition of 1.5 equiv. Cu^{2+} and 15 equiv others metal ions in DMSO/HEPES (1/9, v/v, pH = 6) buffer solution under sunlight and the UV lamp

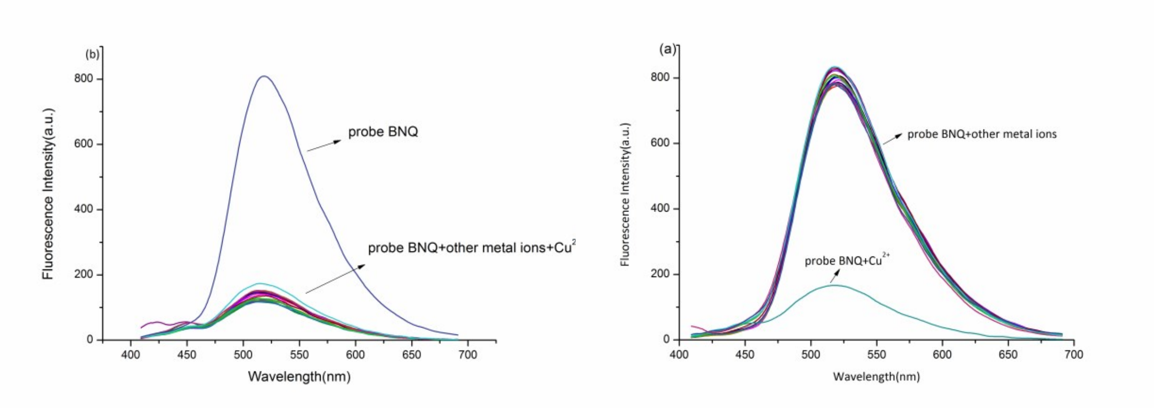

Fig. S10 (a) Fluorescence spectra of BNQ (5 μM) in the presence of other ions (15 equiv.) in the absence of Cu^{2+} (1.5 equiv.). **(b)**. Fluorescence spectra of the probe BNQ (5 μM) in presence of other ions (15 equiv.) after the addition Cu^{2+} (1.5 equiv.).

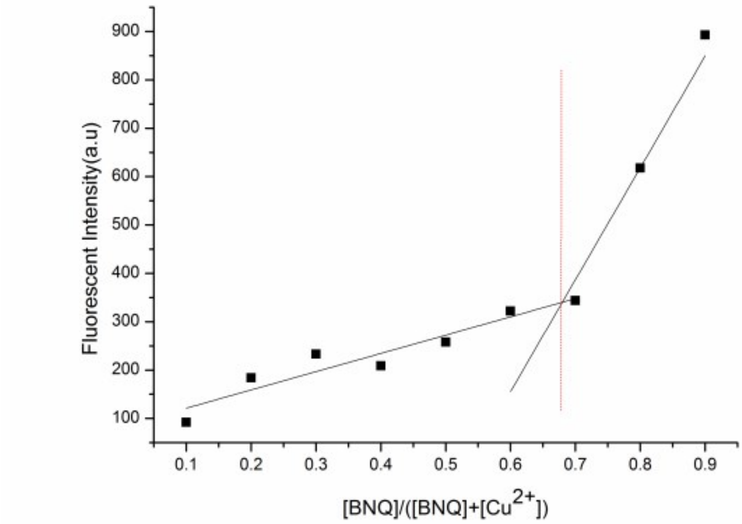

Fig. S11 Job-plot for calculating the stoichiometry between probe BNQ with Cu^{2+} in DMSO-HEPES buffer solution (1/9, v/v, pH=6.0), the total concentration of BNQ and Cu^{2+} was 20 μM.(λ_{ex} = 390 nm).

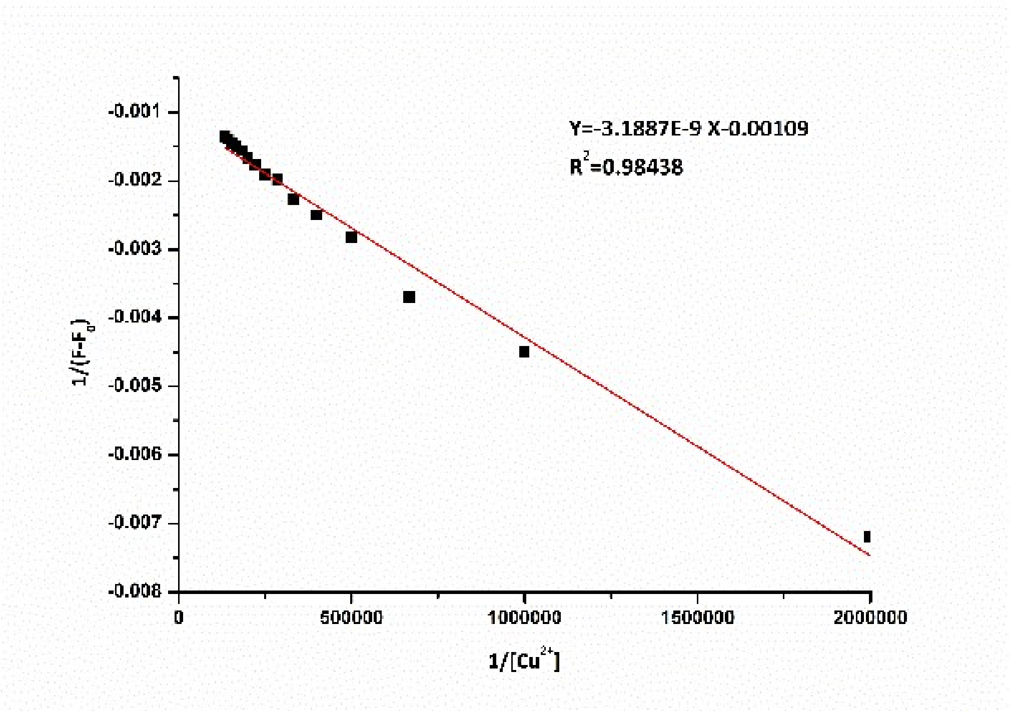

Fig. S12 Benesi-Hildebrand plots (λ_{ex} = 390 nm) of 1/(F-F_0) wersus 1/[Cu^{2+}] for the association base on a 2:1 between probe BNQ and Cu^{2+}.

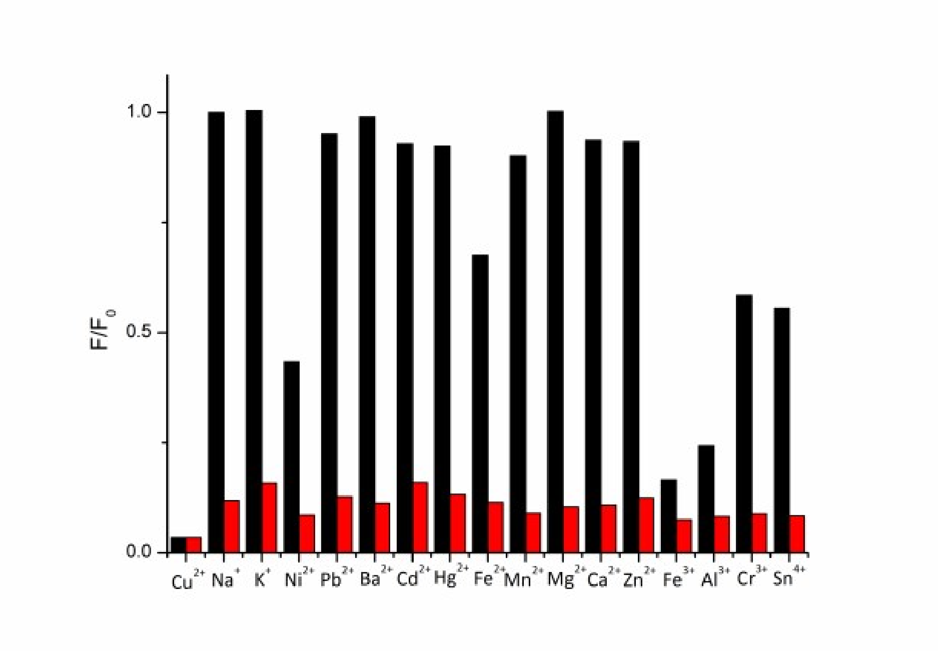

Fig. S13 Y-axis repesents the variation in the fluorescence ratio (F/F_0) at 523 nm. Fluorescence intensity of BNQ2 (5 μM) in the presence of other ions (15 equiv.) in the absence of Cu^{2+} (1.5 equiv.) (black bar). Fluorescence intensity of the probe BNQ2 (5 μM) in presence of other ions (15 equiv.) after the addition Cu^{2+} (1.5 equiv.) (red bar). (λ_{ex} = 389 nm, slits: 5/5, DMSO/HEPES = 1/1).

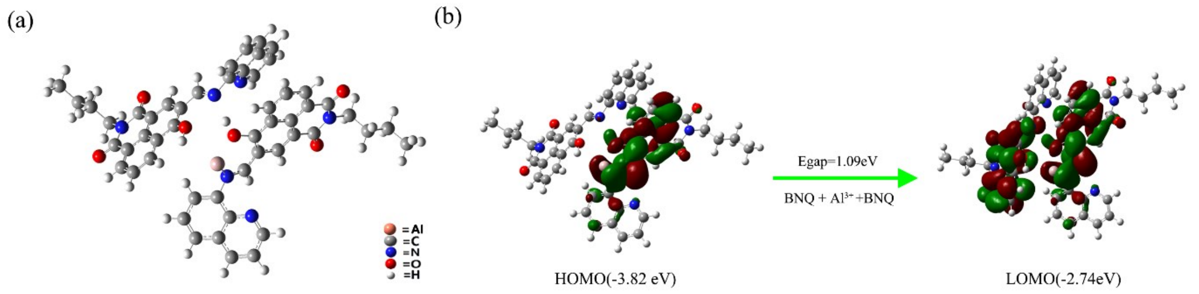

Fig. S14 (a) DFT optimized structures of BNQ+Al^{3+} complex. **(b)** Molecular orbital profiles of BNQ+Al^{3+} molecules

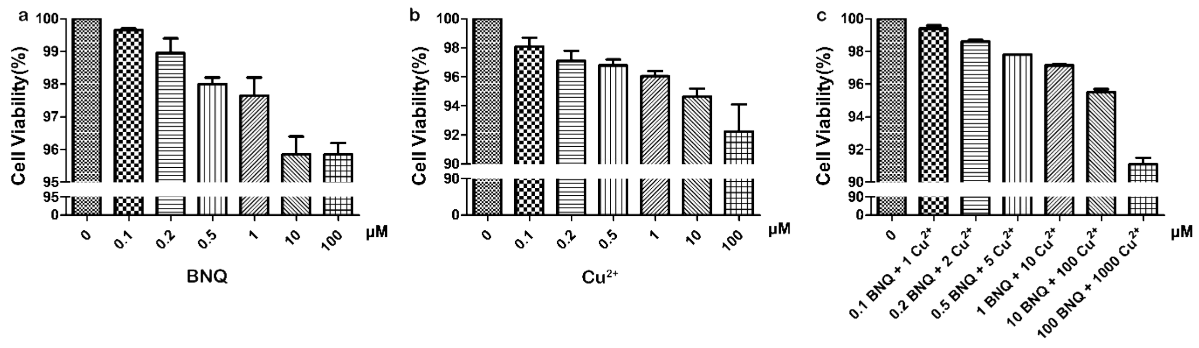

Fig. S15 Cytotoxicity of probe BNQ(a), Cu^{2+}(b), BNQ+Cu^{2+}(c) in Hela cells. Cells were treated with different concentrations of probe BNQ /Cu^{2+}/BNQ+Cu^{2+}at 37 °C for 4 h and cell viability assay were determined by MTT assay
